# Supplementary material for: Differential Modulation of Brassinosteroid and Ethylene Signalling Systems by Native and Constitutively Active Forms of the AtCPK1 Gene in Transgenic Tobacco Plants Under Heat Stress
Source: Plants (Basel). 2025 Mar 26;14(7):1032. doi: 10.3390/plants14071032 (PMC11990407; doi:10.3390/plants14071032)
Supplement: Supplementary file 1 [file plants-14-01032-s001.zip › Supplementary Table S1. Primers pairs; Supplementary Section S1.pdf]

Differential modulation of brassinosteroid and ethylene signalling by native and constitutively active forms of the *AtCPK1* gene in transgenic tobacco plants under heat stress

O.A. Tikhonova[1], V.P. Grigorchuk [1], E.V. Brodovskaya[1], G.N. Veremeichik[1]*

[1]Federal Scientific Centre of the East Asia Terrestrial Biodiversity of the Far East Branch of the Russian Academy of Sciences, Vladivostok, 690022, Russia

*Corresponding author

Address: Galina N. Veremeichik, Federal Scientific Centre of the East Asia Terrestrial Biodiversity FEB RAS, Vladivostok, 690022, Russia. E-mail: gala-vera@mail.ru,

Supplementary Table S1. Primers pairs.

Gene	GenBank № (*N. tabacum*)/Uniprot ID (*A. thaliana*)	Forward	Reverse	Product size, bp
Housekeeping genes				
Actin	XM_016609967	CTCCAAGCAGCATGAAGATTAAG	GACTCGTCGTACTCTGCCTTTG	134
EF1α	AF120093	ACCCTGACAAGATCCCCTTT	TTTCGAGCATGTTGTCACCT	160
BS biosynthesis				
CYP450-85A1	NM_001325207/A0A1S3X3V6	GGAGATGGCTGGATAAGAG	CAAAGTAATGAAGAAATGTAGAA	123
CYP450-90A1	XM_016614025/A0A1S3ZYX3	TGGAGGTGGCAGAGTAATG	CTAAATGGTGAAGGAAAACA	130
CYP450-90B1	XM_016600071/A0A1S3YUB2	GTGGAGATGGCAGAATGAAC	CGTAAGGGAAGGCAAAAGG	230
DSR	XM_016624832/A0A1S4AUY8	GCTGGGGATGCTTAGTATGG	GTGGTAGTAGTGTATGAGGC	238
BS signalling				
BZR	XM_016578743/A0A1S3X2R9	CTTTCCTTCCCTCATCTACAC	GCTTCACCATCCCTCCTGC	300
BRI1-X2	XM_016627480/A0A1S4B2B5	GCTCCGAACCGAACAAACC	GTAAAGCGTCCTGTCGTAATG	200
BRI1	XM_016652863/A0A1S4D4Z4	GAAGAAGAGGTGGAGCAGC	GAAGTGGAGTCGGCTATTATC	200
ET biosynthesis				
ACS6	NM_001326220/AT4G11280	GTTGTTCTTTTCATTGTTCTG	GTTTGCTCCTACTGCATTGTTTC	151
ET signalling				

ERF1	NM_001326142	CAGAGGTGTTAGACGACGG	CAGCCTTTCTTCTCCGTTTAG	360
ERF2	NM_001325036	GCACGGGTTTGGCTAGGGAC	GCTTTTCTCCTCCGTTTCG	230
ERF3	NM_001325253	CAGAGCCCAAGTCACAGCAG	CAACTCCCGCCCGCCACAAAG	247
ERF4	NM_001325499	GGGGACTCGGGTGTGGCTAGG	GGACTCTTTCTTCTCTTTTTAC	240
ERF5	NM_001326275	TGGAGAAAGTCAAGAAATCAC	ACGAATGTTGTGATGAAGTC	220
ERF14	XM_016584910/AT3G23220	GCTCTTCACATTTTTATACTG	CCTCTTGTTTTGCTCATCAC	114
DREB				
DREB2A	XM_016596295/O82132	GATGATTCCTCGTTGCCTAC	TTATCTTGTCCACTCTTCATTG	278
DREB2D	XM_016656435/AT1G75490	CCTCATCTCTACAACAAACAG	CTGAAACGAAACTAGATCTTG	246

Supplementary section 1. Amino acids sequences used in the phylogenetic analisys.

>AtERF095

meriesyntnemkyrgvrkrpwgkyaaeirdsarhgarvwlgtfntaedaaraydraafgmrgqrailnfpheyqmmkdgpngshenavassssgyrgggggddgreviefeylddslleelldygersnqdncndanr

>NtERF14

MDEEPSHGRKMETKNGGSGREIKYRGVRRRPWGKFAAEIRDSARQGARVWLGTYNTAEDAARAYDRAAYSMRGHLAILNFPEEYNLPSSSSHFYTGSSSSREVLEFECLDDKLLEELLDCDEQNKRK

>AtERF49/DREB2D

MSSIEPKVMMVGANKKQRTVQASSRKGCMRGKGGPDNASCTYKGVRQRTWGKWVAEIREPNRGARLWLGTFDTSREAALAYDSAARKLYGPEAHLNLPESLRSYPKTASSPASQTTPSSNTGGKSSSDSESPCSSNEMSSCGRVTEEISWEHINVDLPVMDDSSIWEEATMSLGFPWVHEGDNDISRFDTCISGGYSNWDSFHSPL

>AtDREB2A

MAVYDQSGDRNRTQIDTSRKRKSRSRGDGTTVAERLKRWKEYNETVEEVSTKKRKVPAKGSKKGCMKGKGGPENSRCSFRGVRQRIWGKWVAEIREPNRGSRLWLGTFPTAQEAASAYDEAAKAMYGPLARLNFPRSDASEVTSTSSQSEVCTVETPGCVHVKTEDPDCESKPFSGGVEPMYCLENGAEEMKRGVKADKHWLSEFEHNYWSDILKEKEKQKEQGIVETCQQQQQDSLSVADYGWPNDVDQSHLDSSDMFDVDELLRDLNGDDVFAGLNQDRYPGNSVANGSYRPESQQSGFDPLQSLNYGIPPFQLEGKDGNGFFDDLSYLDLEN

>NtDREB2A

MPSDYSERNQKMAILDQAPKMTSLPLDCSRKRKSRSRRDGTKNVEETLAKWKEYNQKLDCVDDEGKTVRKVPAKGSKKGCMKGKGGPENSRCNYRGVRQRTWGKWVAEIREPNRGSRLWLGTFGTAIEAALAYDEAARAMYGPSARLNLPNYPSSKESSKDDSSWATTSASDSTAGSSLSEVCPAAEQKGISEIKFEDGEGESRIDGVTTAIHEVSTPLTSEKHEAKSKMGVVEAKEEPRSIESINQDMLKSGRDYLDNLNWDELFDVEELLGMLDSIPAGAPAFMQDFGSIAGQKEQYDAYNNNQLSNSSFQHQNADLKLLGGTQQMEQQAPIAVDYGFDFLKPGREEDLNFSLDDLALMDLDSELGV

>XP_016462903.1

MSSHILHRKQKRRRNGCDSLEEILLKWKNHYQELNSSIEDVQVKKKRKIPVKRSRKGCMRGKGGPENSGCIYRGVRQRTWGKWVAEIREPVYNSGRFKTSGKRLWLGTFSTSVDAALAYDEAAKVMYGSNAMLNFPDYCIQNDSSSIVSIARTSSLESTDQSSVDHEDSGAEDAKIRVDQSTFATSVVTADEKQRSCCCLTEKSDVMPEEDYENELNDSGCSSRIDFKPPNYCVKVETPIKEEIEKDEFVHDNDLERLKSYEVSNSLHIMNEETTDVKPHDLNILQEQPLDFRSTENPSEDFCKRLEYMEHWLMEDDCSTEATKVPDTFCLTKNHDEDNSFQKFLEESFDFKPIMVSQESAEFNYVKTEEQFDCTYNQQIDQQTDGIISNQADWNSSISWQPEDSIKDLSVFNFDFDI

>NtDREB2D

MSSTYSVERKTKKITQATSRKGCMRGKGGPENASCTYKGVRQRTWGKWVAEIREPNRGAR LWLGTFENSYDAAVAYDAAARKLYGADAKLNLPHLYNKQAQAQVQAHNSRPNTMGLDNAGGP VSAPPTCNPAAVYNVASPSTWSMGNDHTSFYFNNDFGNNNFSTGDQDLVSFQFLDINQTAEEFDEN NINQNEGIGGEMWRDLNMNLPEIDDSSIWEEAKATTSFQEAVSDPGIYACNLDDGINYPPWC

>NtERF5

MSSNSSPLEIDTSFSHSNFFFLQDQSPILQWDDDLFFNDPWFDDDQSPIIPCNSEKDENHQVFE ESSDNTIMSKGSSHGQELEEVTSQEEKEKEEEEKHYIGVRKRPWGKYAAEIRDSTRNGIRVWLGTF DTAEEAALAYDQAALSMRGPWSLLNFPLEKVKKSLEKIEYSCKDGLSPAAVLKATHKTRRVKHKR SSRKKKNKETHNVIVFEDLGAELLEELLMTSSQHSCRRD

>NtERF1

MNQPIYTELPPANFPGEFPVYRRNSSFSRLIPCLTETWGDLPLKVDDSEDMVIYTLLKDALNV GWSPFNFSAGEVKSEQREEEIVVSPAETTAAPAAELPRGRHYRGVRRRPWGKFAAEIRDPAKNGAR VWLGTYETDEEAAIAYDKAAYRMRGSKAHLNFPHRIGLNEPEPVRVTAKRRASPEPASSSENSSPK RRRKAVATEKSEAVEVESKSNVLQTGCQVELLTRRHQLLVS

>NtERF2

MYQPISTELPPTSFSSLMPCLTDTWGDLPLKVDDSEDMVIYGLLSDALTAGWTPFNLTSTEIK AEPREEIEPATIPVPSVAPPAETTTAQAVVPKGRHYRGVRQRPWGKFAAEIRDPAKNGARVWLGTY ETAEEAALAYDKAAYRMRGSKALLNFPHRIGLNEPEPVRLTAKRRSPEPASSSISSALENGSPKRRR KAVAAKKAELEVQSRSNAMQVGCQMEQFPVGEQLLVS

>NtERF4

MASPQENCTTLDLIRQHLLDDNVFMEHYCPQPILYSQSSSSSESLNSIASELNNETFSFEPTLK YADTAQSSNLDISSFFNNSKTEFDSFEFETKPNVSAARISSNSPKQTSFKERKPSLNIAIPMKQQEVVQ KVEVVPTEKKHYRGVRQRPWGKFAAEIRDPNRKGTRVWLGTFDTAIEAAKAYDRAAFKLRGSKAI VNFPLEVANFKQQDNEILQPANSGRKRMRETENEEIVIKKEVKREERVPAAAAPLTPSSWSAIWEGE DGKGIFEVPPLSPLSPHMAYSQLVMI

>NtERF3

MAVKNKVSNGNLKGGNVKTDGVKEVHYRGVRKRPWGRYAAEIRDPGKKSRVWLGTFDT AEEAAKAYDTAAREFRGPKAKTNFPSPTENQSPSHSSTVESSSGENGVHAPPHAPLELDLTRRLGSV AADGGDNCRRSGEVGYPIFHQQPTVAVLPNGQPVLLFDSLWRAGVVNRPQPYHVTPMGFNGVNA GVGPTVSDSSSAVEENQYDGKRGIDLDLNLAPPMEF

>NP_001292874.1

MKGKGGPDNTQCGYRGVRQRTWGKWVAEIREPNRVDRLWLGTFPTAEDAARAYDEAAR AMYGDLARTNFPGQDATTSAQAALSSTSAQAAPTAVEALQTGTSCESTTTSNHSDIASTSHKLEAS DISSYLKEKCPAGSCGIQDGTPIVADKEVFGPLEPITNLPDGGDGFDIGEMLRMMESDPHNAGGADA GMGQPWYLDELDSSVLESMLQPEPEPEPEPFLMSEEPDMFLAGFESAGFVEGLERLN

>XP_049405102.1

mdqqllptnf pvdfpvyrrn ssfsrlipcl tetwgdlplk vddsedmviy gllkdalsvg
wspfnftage vksepreeie svpefvpspa ettaaratei pkgrhyrgvr qrpwgkfaae
irdpakngar vwlgtyetae eaaiaydkaa yrmrgskahl nfphriglne pepvrvtakr
raspepasss engsmkrrrk avrkcdgeve srsssvmqvgc qieqltgvhq llvs

>XP_059317983.1
mdqpistelp ptnfpvyrrn ssfsrlipcl tetwgelplk vddsedmviy tllkdaltag
wspfnftade vkpeiltspa lttvsppltt aapavlpegr hyrgvrkrpw gkyaaeirdp
akngarvwlg tfetdeeaai aydkaayrmr gskahlnfpn riglnepepv rvtgkrrags
tlsepdgsqk rrrkavgcqv eqltsghqll vs

>XP_060203075.1
myqpistela ptsfsslmsc lteswgdlpl kvddsedmvi ygllqdalni gwtplnltsi
evkaepreet epatspvpsv aptaetttar avvqpkgrhy rgvrqrpwgk faaeirdpak
ngarvwlgty etaeeaalay dkaayrmrgs kallnfphri glnepepvrv tvkrrsaepa
sssissases gspkrrrkav vakqaelevq srpnvmpvge qllvs

>NP_001316388.2
myqlptstel tffpaefpvy crsssfsslm pclteswgdl plkvndsedm viygflqdaf
sigwtpsnlt seevklepre eiepamstsv spptvapaal qpkgrhyrgv rqrpwgkfaa
eirdpaknga rvwlgtyesa eeaalaydka afrmrgtkal lnfphrigln epepvrvtvk
rrlsesasss vssasesgsp krrrkgvaak qaelevesrg pnvmkvgcqm eqfpvgeqll
vs

>XP_058207514.1
mygcecapvi lpanntvefp ayrrsssfss lipclsetwg dlplkvddse dmvvygflrd
avsvgwtpfn itatnvkiep rdemepattm aqlvapqvke epvvspqkgr hyrgvrqrpw
gkfaaeirdp akngarvwlg tyetaeeaal aydraayrmr gsrallnfph rigsnepepv
ritakrrspe pstsvsesgs pkrrkrgvta gevevesqsn afqvecqmrq lpvgeqllvs

>XP_055824772.1
mavkdkavkg gnvkvnngvk evhyrgvrkr pwgryaaeir dpgkksrvwl gtfdtaeeaa
kaydtaarkf rgpkaktnfp faseinnntq spcqsstaes ssgetgvhap htpqeldltr
rlgavvaegg ragfpifqqq ptvavlpngq pvllfdsmwr pgvvnggqmp ypvapvamef
agagagvvsc vsdlssvvee nqcvakkgld ldlnlappme v

>XP_059282506.1

mavkdktsnn vkangvkevh frgvrkrpwg ryaaeirdpg kksrvwlgtf dtaeeaakay
dtaarefrgp kaktnfplps enqspshsst vesssgetge argvhaphap leldltrrlg
eggvcaggng ypifhqqptv avlpngqpvl lfdsmwrpgv vnrgqlpyqv apvamefngv
gvgagavpsv sdsssvveen svvgkrgldl dlnlappmev

>GMD36789.1
mavkgregav kggagkvngi kevhyrgvrk rpwgryaaei rdpakksrvw lgtfdtaeea
araydaaare frgakaktnf hspsenhsps qsstvessgs etaghapqfp leldltrrlg
saeaagvrsv nnnnntfqff hpqpavavlp ngqpvllfet lwrpgavsrp lpdqfeaapa
ipskrpalsd sstfsvveen nfvgagagva ekrlnldlnl apptea

>XP_059305018.1
mgspqencsl dlirehlldd vpfmdyycsq itqtssssqs ldssvnnnss esldrtfsfe
ttfnlaessn vdissffsss ktefdcffel etkpnvsaar itsnspkqts fkerkpslni
aipakpivqk vevamesekk hyrgvrqrpw gkfaaeirdp nrkgtrvwlg tfdtaidaak
aydraafklr gskailnfpl evanfkqqhd veiqpqpvns grkrvreten eeqevikkev
kieeeravta apltpsswsa vwdcgngkgi fevpplspls phmsysqlvm i

>MCD7460968.1
massqetcst ldsirehlld ddivfmehyk ntlysqsfes ldqssfsfet tfnscantad
qslnleissf fnssetefdc ffevetkpnv savnspkrrs fkerkpslni aipakpvaqk
vevlsenekk hyrgvrqrpw gkfaaeirdp nrkgtrvwlg tfdtavdaak aydraafklr
gskailnfpl evanfkqqnd vevpqpqvss sskrvreteq liikkerkie eeravptgaa
apltpsswst iwdcgngkgi fevpplspls phmgysqllm i

>NP_001311615.2
mgspqencsf dmirqhlddi slmeyycpen tlysqscesl dqtsvsfets snlddissff
sssktdfgcf efetkpitsa atsissnspk krsfnerkps lniavpvkpv vvqkvevvre
kkhyrgvrqr pwgkfaaeir dpnrkgtrvw lgtfdtavda akaydraafk lrgskailnf
plevanfkqq nstvevpqqv nssgskrare keelainkem kieeeervaq tapltpsnws
tiwdsgngkg ifevpplspl sphmsysqlv mi

>XP_011076436.1
maspdessal eiirqhlldd saflqtycss pvfdsdnsqi srtssnsssv lseltnssts
sssveqsssv fmpvftatsn lefsgffrse aapeflefet kprilcsssd pmkqknfser
kpslniaipt vskkvewdqn fdasfkvqkv aerkveansa drrhyrgvrq rpwgkfaaei
rdpnrkgtrv wlgtfdtave aaraydraaf klrgskailn fpleigtsnp lpqneaapaa

gcrkrktewe ceerekkevk rreeirevkt eseataavpl tpsswtavwd ggdgkgifev
pplsplsphp slgysrlmvi

>XP_049366414.1
mssnssplei dtsfshsnll ffqdqspwss ndlffndpfv dideyppiii pcnhqdivve
ssntttttsk asshnshhdq eevtsqekkq eddqqkhyig vrkrpwgkya seirdstrng
irvwlgtfdt aeeaalaydq aalsmrgpws llnfpiedvk kslekieysc kdglspaavl
kathktrrvk hkrsskkknn knlenvfvfe dlgvelleel lmtss

>XP_027076366.1
mdhvtksfag asssskeing eeftlartra kedhygkekh yvgvrkrpwg kfaaeirdst
rngirvwlgt fdtaeeaala ydqaalsmrg pvaalnfpae rvheslqemv lfscedglsp
aaalkevhkm rsmskrrgkr kqlvkgddlv vfedlgpdll dellsesess rck

>PHU17989.1
mssnssspple idtsfsqsnf fffqeqsptt pwssnndlff ndpfvdddqy spiiipcdye
kdhhhdqdlv vessnttits kvssqnshhd qeevtsqekk qedeqeekhy igvrkrpwgk
yaaeirdstr ngirvwlgtf dtaeeaalay dqaalsmrgp wsllnfpiek vkkslekiey
sckdglspaa vlkathktrr vkhkrksrkk knnlenvlvf edlgvellee llmtps

>XP_004247924.3
meqdpsnerr tnggggeiky rgvrrrpwgk faaeirdsar qgarvwlgtf ntaeeaaray
draaysmrgh lailnfpeey nlpsssshfy sagsyssssm asssssssr qvlefeyldd
klleelldcd eepnkrk

>XP_021667608.2
mqntqmdedr amareeatgr evkyrgvrrr pwgkfaaeir dstrqgarvw lgtfntaeea
araydraaya mrgqlailnf pneytlasgg sgsvtassss sspsmqknvi eleclddrll
eemldqeeqk skkk

>XP_059317949.1
maildqasnm isipmdytrk rksrsrkdap knvaetiakw kevneklnss vrkvpakgsk
kgcmkgkggp enarckyrgv rqrtwgkwva eirephrgnr lwlgtfqtai eaalaydeaa
ramygpcarl nlpnytsske svtddsslpt isqsdsttas sfsevcpadd krgrtnilks
esrvddtric sseagtplss vmeeakdeak galdtrevre ikeepvtfdy dvqksgqdnl
enfgwddemf dvdvllsmlg stpldasapn qdgsfppkqe qyaydpsyrm qsaaydnnqf
pyqsyqleda dnqfsnpsyq ldnadgktfe glqqmdqqap navdydfdfl kpgrqedfnf

cldelglldf q

>ADZ15315.1
mavldrtsnm mlpmdytrkk ksrsrkdapk nvaetlakwk evnekldacd ddgrkpvrkv
pakgskkgcm kgkggpdngr ckyrgvrqrt wgkwvaeire phrgrrlwlg tfdtaieaal
aydeaaramy gpcarlnlpd yyasskessk ddsslptvsr sdsntassfs evcpagdmmr
granvpaarh edrsieidga rtgsneigtp lsslreeaed etkevsdkse tftplsslre
qaedeakqvl dksetfeikd epaacsydsw digqedlgnf clddemfdvn ellgmmdstp
vdasapsqdv gfvppkqeqy aydpsyqlhs aaydanqlsn payqldnadd qfsnplyqle
nagvdtlegl qqmeqqspie veydfdflrp grqedfhfcl deldvldf

>QHW05977.1
miaqniefqr ntmmglldqa snltslpldy srkrksrsrr ngsksvsetl akwkeyndkl
dssdegakpv rkvpakgskk gcmkgkggpe nshcnyrgvr qrtwgkwvae irepnrgsrl
wlgtfptarq aamaydeaak amygpcarln lpngvslses gkestslptt sgsdstttsi
qsevcasedl mvnldapkvk heddnrlsti csvstpmsvv kdkikeepvd fnveselgyg
qnypqnftyd demfdvdell galgstsvhd lgslgqffdy nkfngempsd lsyqlqnpda
kllgslhhme qvpsavdygf dflrpgrqed hnfalddfgy lnldyysdlg l

>XP_027768082.1
mashvaqrkv krrrngsdsi eeillrwrnl nqevnsnneq vkkkrkspgn gsnkgcmpgk
ggpensgcky rgvrqrtwgk wvaeirepvy isgqykskgk rlwlgtysta gdaavaydea
akvmygsnai lnfpnscdss sngnitrtss gqssidheel vvddekktei esdlkdddgd
ggvvvnmdls ydyanhgspa cswteeelev iteensemel tnlecdsrff pksqkscvev
erpimeeeid edelvhnnvs ntidveptvm fskddfsrld eicnsndqiv lqdmdfrsce
nlnedvstrl eymehflmdd ncsmeaanis diicltenhd eafdfqrfle esfdfkpmae
lnyakneeqf dctyaynqqi dlqnsdtnse irsdgirkek nlhgfrlddf gasnswqpee
niedlsmcsf dfdissyind in

>KAF3624146.1
mssnftesvd dqgrpkkqtq akltqatsrk gcmrgkggpe nasckykgvr qrtwgkwvae
irepnrgarl wlgtfdnsyd aavaydaaal klygaeakln lphlynnpaq aqeeinsnsg
pitinptpap apapvvynva spstwsvgnd pslyfdndyd insfatsddp isidfmglmd
ttdheefaek nnnqisqglg gemwtdekvn nqisqglgge mwtdlnmlpe iddssiweea
eattsfqeav indpgvngcn ldgginfppw c

>XP_027148674.1

meadrsgngn grpgkkpaqa ssrkgcmrgk ggpenascay kgvrqrtwgk wvaeirepnr
gsrlwlgtfd tsheaaiayd aaarklygta aklnlphlyr rpqhipvyst lntttpidtg
genqtglrpv pfqqspfrnf asssnssypp qcqpnerlff qtetnatttg ylagtttagv
tsvpndnnnf vddngneave seegtmrgmw nklnanlpai ddssiwaeaq aaspfqaavt
dpgifggnfe dsnypwn

>XP_013717769.1
mehrasntnq vkyrgvrkrp wgkyaaeird sanhgarvwl gtfntaedaa raydrsafsm
rghkailnfp heypmmrdgp sgdgenagas lssefserdg gggkeviefe ylddsllldel
lecgesynqd vwynatvn

>XP_042970592.1
msamedkegg evkyrgvrrr pwgkfaaeir dstrhgarvw lgtfntaeea araydrsaya
mrgslailnf pneyplasgg ssapssssaa ssssgrgnmg ergrsehrqq vfefeclddk
vledlldsee kknkk

>XP_023637359.1
mavydqsgdi nktqldtsrk rksrsrrdgt tvaerlkrwk eyndtveeas tkkrkvpakg
skkgcmkgkg gpengrcsfr gvrqriwgkw vaeirepnrg srlwlgtfpt aeeaasayde
aakamygpla rlnfppsdgs evtstssqse vctvetpgca hvktedadce skplfgeanp
mchlesgadv nkkdvkadvd wlsefennyw sdvlkekeke kkeqeivetc kqqpdslsva
dygwpndldq shwdpsemld vdellgding dlftglnhdq yagnnvdigs fkperrqggy
qslqsldygl ppfqleakdg ngffddlsfl dlen

>KAJ0266348.1
mavydnsgdi nrteldtskk rksrsrrdgi tvaerikmwk eyndtieesp skkrkvpakg
skkgcmkgkg gpensycsfr gvrqrtwgkw vaeirepnrg srlwlgtfpt aeeaasayde
aakamygpla rvnfpqssvs dvastssrse vctveapsva ahvkredgdc esrpffadak
pledvkrdlk vddpgadwls efeqeywtgl leekakqkeq eivaggsvqk gqpdslsvsd
ygwpadvyqn qwdssemfdv sellgdlngd iftgtdkdqc lgvnvgggls eperqriglp
plqslglppp qheaqdgnnf fdpsfldvkn

>KAJ4911663.1
mqdnatmscc hlpitsadwf sryhpqtqfl rfiavvdvsk qsqktlqivs nrseplkiks
wfqlafavag gcgritreed akrrihrrsk eirtstlddq trdrfdslrk etelipiisp
idcvvsgkkm avydhsgdin stqldtsrkr ksrsrrdgtt vaerlqvwke yndnieaasp
kkrkvpakgs kkgcmkgkgg pensrcsfrg vrqriwgkwv aeirepnrgs rlwlgtfpta

eeaalaydea arvmygpsva rlnfpqksvs dvistssqse vctagtsgrv hvktedadcd
serflgeanp lengvgeikn dvkvdapstd wlgefeqkyw sevleekekq kqkqkkqvet
cekqpdslsv adygwpedld qtqwdpsemf dvsellgdln gdiytgsnqs qypwdneaqq
tglqgldsgy elpplelevq dgnelfdlsf ldlek

>NP_001185352.1

MDSSCIDEISSSTSESFSATTAKKLSPPPAAALRLYRMGSGGSSVVLDPENGLETESRKLPSSK
YKGVVPQPNGRWGAQIYEKHQRVWLGTFNEQEEAARSYDIAACRFRGRDAVVNFKNVLEDGDLA
FLEAHSKAEIVDMLRKHTYADELEQNNKRQLFLSVDANGKRNGSSTTQNDKVLKTREVLFEKAVT
PSDVGKLNRLVIPKQHAEKHFPLPSPSPAVTKGVLINFEDVNGKVWRFRYSYWNSSQSYVLTKGWS
RFVKEKNLRAGDVVTFERSTGLERQLYIDWKVRSGPRENPVQVVVRLFGVDIFNVTTVKPNDVVA
VCGGKRSRDVDDMFALRCSKKQAIINAL

>Solyc05g009790.2.1

MEGSISSIDQESTTSDSLSIAPAASSSTMIKSSTTIKLPPESGLCRMGSGTSVIIDAENGVEAESR
KLPSSRYKGVVPQPNGRWGAQIYEKHQRVWLGTFNEENEAARAYDIAAQRFRGRDAVTNFKPLLE
NQESDDMEIAFLNSHSKAEIVDMLRKHTYIDELEQSKRLFGFTKDGMIKRKDGLVISSFFGSTNDKV
NCKAREQLFEKVVTPSDVGKLNRLVIPKQHAEKHFPLQNGNNSKGVLLNFEDLNGKVWRFRYSY
WNSSQSYVLTKGWSRFVKEKNLKAGDIVSFQRSTSGDKQLYIDFKAKNVGNTSMVVTNQVQAQV
QVPLVQMVRLFGVNICKVPANVSNVIILGGFCFEVFTEFGSFVGNNANFSKKLVIYAKRLSGLEEAL
RIRRERELRGSTTATRRPPLRRGRVSQRLPVPDDIPKPPYVGSRELPEISSEHQIHDAEGIARMRAACE
LAARVVDYAGTLVRDPKCTLDVSTQKFICYHLITVAHSASLIPSVTTNEIDKAVHQMIIDAGAYPSP
LGYGGFPKSVCTSVNECMCHGIPDSRQLQDGDIINIDVTVYLNGYHGDTSKTFFCGDVSESIKRLVK
VTEECLHYGIAVCRDGALYRKIGKRISEHAEKFGYGVVDRFVGHGIGTVFHSEPLIFHHQPILTLGST
ECVTWEDNWTTLTADGSPAAQFEHTILITKTGAEILTTY

>ONM39018.1

MDSASSLVDDTSSGGGGGASTDKLRALAVFAAASGTPLERMGSGASAVVDAAEPGAEADS
GSGAAAVSVGGKLPSSRYKGVVPQPNGRWGAQIYERHQRVWLGTFAGEADAARAYDVAAQRFR
GRDAVTNFRPLADADPDAAAELRFLASRSKAEVVDMLRKHTYFDELAQNKRAFAAASASAATASS
LANNPSSYASLSPATATAAAREHLFDKTVTPSDVGKLNRLVIPKQHAEKHFPLQLPSAGGESKGVL
LNLEDAAGKVWRFRYSYWNSSQSYVLTKGWSRFVKEKGLQAGDVVGFYRSAAGADTKLFIDCKL
RPNSVVVASTAGPSPPAPVAKAVRLFGVDLLTAPATAAAPAEAVAGCKRARDLGSPPQAAFKKQL
VELALV

>NP_178088.2

MAKVSGRSKKTIVDDEISDKTASASESASIALTSKRKRKSPPRNAPLQRSSPYRGVTRHRWTGRYEAHLWDKNSWNDTQTKKGRQVYLGAYDEEEAAARAYDLAALKYWGRDTLLNFPLPSYDEDVKEMEGQSKEEYIGSLRRKSSGFSRGVSKYRGVARHHHNGRWEARIGRVFATQEEAAIAYDIAAIEYRGLNAVTNFDVSRYLNPNAAADKADSDSKPIRSPSREPESSDDNKSPKSEEVIEPSTSPEVIPTRRSFPDDIQTYFGCQDSGKLATEEDVIFDCFNSYINPGFYNEFDYGP

>XP_004235883.1

MAKTSKSNTTSTSSSSSSNKCDSKAKRSNKIDGNAIGKVKRTRKSVPRDSPPQRSSIYRGVTRHRWTGRYEAHLWDKNCWNETQNKKGRQVYLGAYDDEEAAAHAYDLAALKYWGQDTMLNFPIMTYENELKEMEGQSKEEYIGSLRRKSSGFSRGVSKYRGVARHHHNGRWEARIGRVFGNKYLYLGTYATQEEAATAYDMAAIEYRGLNAVTNFDLSRYIKWLRPSDQTNNDNTIINPEPNPNPNPNDIHLMPNTKDDTNFTQQQQQISGCDVTVAALPHPGGGAATSSAALELLLQSTKLKEMLERRSEVIECPETPPEPDRPRRSFPDDIQTYFDCQEPSSFIEEHDIIFGDLDSLTLPMFQCELIN

>XP_008651355.1

MARPRKNSAAAANNNNSNTTNAGNAAVDLAARVKPKRTRKSVPRESPSQRSSVYRGVTRHRWTGRFEAHLWDKNSWNESQNKKGKQVYLGAYDDEDAAARAYDLAALKYWGPDTILNFPASAYEAELKEMEGQSREEYIGSLRRKSSGFSRGVSKYRGVARHHHNGRWEARIGRVFGNKYLYLGTYGTQEEAAMAYDMAAIEYRGLNAVTNFDLSRYIKWLRPGAGAAQNPHPMLDGLAQQLLLSPEGTIDGAAFHQQQHDHRQQGAAELPLPPRASLGHTPTTSALGLLLQSSKFKEMIQRASAAESGTTTVTTTSSSSSQPPTPTPTPSPSPPPTPPVQPARDASPQCSFPEDIQTFFGCEDVAGVGAGVDVDALFFGDLAARMELSRHLRTKKMVRHTKQERQQNRQTAPSLF
